# Post-COVID Public Health Surveillance and Privacy Expectations in the United States: Scenario-Based Interview Study

**DOI:** 10.2196/30871

**Published:** 2021-10-05

**Authors:** John S Seberger, Sameer Patil

**Affiliations:** 1 College of Communication Arts & Sciences Michigan State University East Lansing, MI United States; 2 School of Computing University of Utah Salt Lake City, UT United States

**Keywords:** COVID-19, pandemic-tracking apps, privacy concerns, infrastructure, health surveillance, scenario, interview, thematic analysis

## Abstract

**Background:**

Smartphone-based apps designed and deployed to mitigate the COVID-19 pandemic may become infrastructure for postpandemic public health surveillance in the United States. Through the lenses of privacy concerns and user expectations of digital pandemic mitigation techniques, we identified possible long-term sociotechnical implications of such an infrastructure.

**Objective:**

We explored how people in the United States perceive the possible routinization of pandemic tracking apps for public health surveillance in general. Our interdisciplinary analysis focused on the interplay between privacy concerns, data practices of surveillance capitalism, and trust in health care providers. We conducted this analysis to achieve a richer understanding of the sociotechnical issues raised by the deployment and use of technology for pandemic mitigation.

**Methods:**

We conducted scenario-based, semistructured interviews (n=19) with adults in the United States. The interviews focused on how people perceive the short- and long-term privacy concerns associated with a fictional smart thermometer app deployed to mitigate the “outbreak of a contagious disease.” In order to elicit future-oriented discussions, the scenario indicated that the app would continue functioning “after the disease outbreak has dissipated.” We analyzed interview transcripts using reflexive thematic analysis.

**Results:**

In the context of pandemic mitigation technology, including app-based tracking, people perceive a core trade-off between public health and personal privacy. People tend to rationalize this trade-off by invoking the concept of “the greater good.” The interplay between the trade-off and rationalization forms the core of sociotechnical issues that pandemic mitigation technologies raise. Participants routinely expected that data collected through apps related to public health would be shared with unknown third parties for the financial gain of the app makers. This expectation suggests a perceived alignment between an app-based infrastructure for public health surveillance and the broader economics of surveillance capitalism. Our results highlight unintended and unexpected sociotechnical impacts of routinizing app-based tracking on postpandemic life, which are rationalized by invoking a nebulous concept of the greater good.

**Conclusions:**

While technologies such as app-based tracking could be useful for pandemic mitigation and preparedness, the routinization of such apps as a form of public health surveillance may have broader, unintentional sociotechnical implications for individuals and the societies in which they live. Although technology has the potential to increase the efficacy of pandemic mitigation, it exists within a broader network of sociotechnical concerns. Therefore, it is necessary to consider the long-term implications of pandemic mitigation technologies beyond the immediate needs of addressing the COVID-19 pandemic. Potential negative consequences include the erosion of patient trust in health care systems and providers, grounded in concerns about privacy violations and overly broad surveillance.

## Introduction

### Background

The COVID-19 pandemic has raised profound concerns about the potential effects of app-based public health surveillance. On one hand, apps for contact tracing and exposure notification — which we refer to as “pandemic tracking apps” [1] — are potentially useful for pandemic mitigation [2-5] and preparedness for future pandemics [6]. In this regard, such apps are understood to contribute to “the greater good” by helping achieve stable and acceptable levels of public health [6,7]. On the other hand, the same technologies have stoked fears of mass surveillance with an ever-increasing scope [8-12]. In this regard, the relationship between pandemic tracking apps and the long-term greater good is less clear. Even positive assessments of such apps acknowledge their inherently “creepy” nature [13], implicitly underlining a parallel between the restoration of public health and the data-driven economics of surveillance capitalism [14].

Mobile health (mHealth) research is fertile ground for improving quality of life (eg, [15-17]). The relationship between short- and long-term forms of the greater good that may be achieved through the routinization of pandemic tracking apps deserves deeper consideration in terms of the broader quality of life these apps could foster. These considerations include people’s beliefs about the right to privacy in an increasingly technology-mediated world, people’s trust in the health care institutions that provide care to them, and the affective outcomes of such beliefs.

While other work at the intersection of privacy and pandemic mitigation technology has (rightfully) focused on the immediacy of the pandemic [7,18,19], we approach people’s perceptions about pandemic tracking apps with a focus on the long-term implications. Echoing prior work in human-computer interaction (HCI) [1], we adopted a future-oriented lens to analyze how people understand the potential ramifications of app-based public health surveillance after the pandemic. We paid particular attention to the potential effects of such surveillance on everyday sociotechnical conditions (ie, the ways in which social norms and technological capabilities mutually influence each other and thereby shape daily life) [20,21].

We begin by covering salient literature in the sections that follow. Given the disciplinary intersectionality of this work, we discuss related work from several domains.

### Health, Surveillance, and Pandemic Tracking Apps

Individual health, public health, and privacy converge within the field of health surveillance. The history of health surveillance (eg, [22,23]) shows a tendency toward general surveillance [24]. Although the ultimate object of health surveillance is a specific disease, such surveillance necessarily includes the person that carries that disease. Recent work has framed the COVID-19 pandemic as an opportunity for heightened general surveillance [8,12,25]. Such framing is appropriate because of 2 characteristics of technology use at the time of the COVID-19 pandemic. First, pandemic mitigation techniques, such as quarantines and shutdowns, have increased people’s daily reliance on technology [26]. Second, technological solutions to the pandemic are entangled with institutions whose survival is predicated on data-driven “dehumanization” of the user [27] and corporate initiatives that foster people’s resignation to accepting privacy violations as inevitable [28]. Both characteristics are broadly aligned with the data-fueled economics of surveillance capitalism [14], in which people are implicitly operationalized as monetizable sources of data that are trafficked and profited upon through practices such as personalized advertising.

For example, in the United States, Google and Apple have worked together to create an exposure notification infrastructure [5]. The infrastructure these parties have developed is privacy-preserving, but in a short-term way. That is, the proposed and implemented privacy protections focus on limiting the identifiability of data, rather than understanding people’s perceptions of the data collection in the long term. Researchers have argued that the short-term measures taken by Google and Apple may limit the value of the data collected through such an infrastructure [2]. Such an argument implies that app-based data should be squeezed for all the information it might provide. Digital pandemic interventions elsewhere in the world have leveraged similarly large-scale corporate or military platforms [25]. Such collaborations between industry, military, and the government highlight the blurry line between health surveillance and the data-driven objectification of the user [8].

Despite the murkiness of the economic, institutional, and social realms in which pandemic tracking apps function, such apps are clearly aligned with the foundational logic of health surveillance (ie, [22,29-31]) and have well-defined benefits in terms of public health. However, they raise concerns about what is termed as “surveillance creep” (ie, the tendency for the surveillance abilities of a technology used in one context to transfer to a similar technology in a different context, thereby achieving subtle forms of social control [32]). For example, in the case of the Google/Apple collaboration, surveillance creep manifests as the extension of platform-specific surveillance capabilities (eg, user tracking, geolocation) into the domain of public health. Surveillance creep in public health may have a profound impact on the postpandemic everyday lives of users [11,24]. This is particularly apparent in light of medical-technical programs that prescribe smartphones as part of mitigation routines (cf. [33]).

Broad concerns about surveillance creep are manifested in short-term concerns about end-user privacy. By short-term concerns, we mean design-oriented concerns intended to increase the use of apps without sufficiently considering the long-term implications of widespread adoption. For example, recent work has shown that mHealth apps do not typically provide sufficient information about the third parties to whom data access might be provided [34]. Similar work has identified widespread insufficiencies in mHealth privacy policies [35]. However, no work to date has focused specifically on the potential routinization of pandemic tracking apps as infrastructure for public health surveillance. Two necessary areas of inquiry regarding the impact of routinization are (1) people’s beliefs about their rights to privacy in an increasingly technology-mediated world and (2) people’s perceptions of the health care systems that provide care to them.

### Pandemic Tracking Apps as Potential Infrastructure

Pandemic tracking apps may form future infrastructure for public health surveillance. As such, the concept of the “hopeful monster” is relevant to the potential routinization of pandemic tracking apps. The hopeful monster is a technology that aspires to the functional invisibility of infrastructure [36,37]. In this way, the hopeful monster exists as a precursor to a successful technology, where successful technologies are those that become invisible through routinization [38].

However, the potential success of pandemic tracking apps, and therefore the likelihood of their invisibility, remains uncertain. In the short-term, pandemic tracking apps will be considered successful if they prove to be effective in controlling the current COVID-19 pandemic. In the long-term, however, it is naïve to assume that a successful, population-scale mode of health surveillance will be dismantled after the pandemic or used only for its originally intended purpose (ie, surveillance of the COVID-19 pandemic) [39]. Indeed, research is already underway to examine the applicability of pandemic tracking infrastructure for tracking other diseases including HIV/AIDS in the United States [40], the benefits of routinizing app-based surveillance in the form of smart technologies [41], and the development of international interoperability for pandemic tracking apps [42]. Recent work has further highlighted the likelihood of pandemic tracking apps being routinized as future means of pandemic preparedness and mitigation [6]. The effects that such apps have on their users will likely extend beyond the end of the current pandemic.

Technology adoption models have been employed to understand how and under what conditions individuals may adopt pandemic tracking apps [18,43,44]. Once a technology is adopted for use, the satisfaction that users feel when an adopted technology “does what it is supposed to do” fosters continued use [45], particularly in mHealth apps [44,46]. Moreover, people have been shown to approach apps that serve the greater good with less privacy wariness and greater willingness to overlook privacy concerns [6,47]. Assuming that adoption of pandemic tracking apps is effective for mitigating the spread of the COVID-19 pandemic, such apps are primed for continued use in nonpandemic circumstances and become typical means for public health surveillance [1,6].

Data collected from a sample of Amazon Mechanical Turk workers in the United States indicate a generally favorable expectation to continue using pandemic tracking apps for general health monitoring even after the COVID-19 pandemic has been controlled [1]. People demonstrating a collectivist social orientation are more likely to continue using such apps for general health monitoring. Similarly, younger users expect to continue using such apps after the pandemic [1], aligning with their higher inclination to adopt pandemic tracking apps [48].

At the same time, Seberger and Patil [1] found that privacy concerns regarding mobile apps are negatively correlated with the expectation to use pandemic tracking apps as infrastructure for general health surveillance. People who are more concerned about privacy when using mobile apps are less likely to use pandemic tracking apps for general health monitoring *after* the pandemic. However, no such relationship was found between privacy concerns and the perceived benefit or expected use of pandemic tracking apps *during* the pandemic [1]. Given the prevalence of hyperbolic discounting [49] (ie, people’s general inability to judge future impacts of current privacy decisions), it is likely that the relaxation of privacy concerns caused by the immediacy of the pandemic implicitly fosters long-term routinization of pandemic tracking apps.

Apps are designed to be used, and success in meeting user needs fosters their continued use [44,45]. If pandemic tracking apps prove to be successful in the mitigation of COVID-19, then it is possible that they will form the core of a more general, app-based form of public health surveillance. As we begin transitioning beyond the pandemic, it is essential to examine how people understand the sociotechnical implications of such an app-based infrastructure for public health surveillance that extends into the future.

### The Relevance of Privacy to Pandemic Tracking Apps

We consider people’s privacy concerns as a lens through which we can understand their expectations of postpandemic public health infrastructure. Theoretical approaches to end-user privacy concerns vary widely across scholarly disciplines [50]. Recently, there has been an increased focus on the politics of definitions and theorizations of privacy [51]. Whether privacy is understood to be relational [52,53], normative [54-57], calculative [58-60], or affective [57,61], policies and practices derived from such approaches necessarily shape the daily expectations and practices of the worlds in which they are realized. Such expectations and practices are increasingly characterized by the economics of surveillance capitalism [14].

The pervasiveness of end-user privacy concerns that arise in relation to surveillance capitalism [14] has been demonstrated to contribute to digital resignation [28], learned helplessness [62], and security fatigue [63,64]. Digital resignation refers to a social stance toward technology in which people are resigned to the inevitability of negative effects of technology. Digital resignation is thus a form of learned helplessness, which has been demonstrated to arise in relation to negative user experiences. Similarly, security fatigue refers to the taxing effects of maintaining security and privacy over time. It is possible to extrapolate from security fatigue [63,64] to the longer-term fatigue that may arise in the context of an ever-changing ecology of devices, apps, and protocols. Further, privacy researchers often encounter the so-called privacy paradox [65]: People tend to *state* that they are concerned about privacy and *act* as though they are not. Similar to recent work [66], we consider the privacy paradox obliquely through one of its symptoms: fatalistic attitudes toward the inevitability of app-based privacy intrusions.

To frame our work, we drew on research about the experience of creepiness in relation to technology use [62,67]. More specifically, we explored the potential for creepiness in modern platforms (eg, Facebook, Google, Amazon) that are capable of increasing levels of surveillance [20,68,69]. We approached our research in terms of the varied and ad hoc groupings of technologies used in a person’s daily life (ie, assemblages [70]). We further consider 2 privacy-related phenomena: the appearance of hyperfunctional infrastructures [71] and hyperbolic scaling [66]. Hyperfunctional infrastructures work according to their intended purpose but do so in ways that render them visible through the affective conditions they bring about [71]. Relatedly, hyperbolic scaling refers to user tendencies to project the negative characteristics of 1 app or category of apps onto the whole app ecology [66].

### Research Question

Future public health may well benefit from the routinization of app-based surveillance based on the infrastructure resulting from the deployment of apps to track the COVID-19 pandemic. Given the immediacy of triage and the severe impact of COVID-19 on public health, it is tempting to focus exclusively on the short-term need to stabilize public health to an acceptable level. However, improved or stable public health is only one possible outcome of app-based public health surveillance. Other outcomes play out on a longer temporal scale.

The goal of our research was to understand the interplay between perceived public health benefits of pandemic tracking apps and their potential effects on nonmedical concerns (eg, sociotechnical conditions, surveillance capitalism). To that end, we addressed one core research question: How can people’s privacy concerns about pandemic tracking apps improve our understanding of the sociotechnical ramifications of a potential postpandemic technical infrastructure for public health surveillance?

We identified a need to engage in the present work for 2 reasons. First, prior work has shown that people are interested in continuing to use pandemic tracking apps as infrastructure for public health surveillance [1]. Second, people expect such continued use to occur within a broader economy of surveillance capitalism [14]. Given that technology often “moves fast and breaks things” [10,39], the academic research community should devote energy to understanding these possible outcomes before they are realized. We adopted such a stance with the hope of engaging with the broader health-related research community.

## Methods

### Overview

To answer the research question posed in the previous section, we conducted semistructured scenario-based interviews (n=19) during the spring and summer of 2020 with adult participants from the United States. All study procedures were approved by the Institutional Review Board of Indiana University (Protocol #1902443119). We present the research according to the Standards for Reporting Qualitative Research guidelines [72].

The pragmatic nature of our research called for bottom-up qualitative analysis [73]. Building on prior human-centered work in HCI, we engaged in inductive analysis to surface beliefs about the possible ramifications of developing infrastructure for public health surveillance on the back of pandemic tracking apps. Recent work has highlighted the value of such inquiries for understanding the user experience of mHealth devices [74]. We analyzed interview transcripts by engaging in the inductive and constructivist process of exploratory pragmatic research using reflexive thematic analysis [75].

### Scenario Development

The use of scenarios is common in privacy-related research (eg, [1,66,76-78]). We developed a scenario that describes a fictional pandemic tracking application linked to a smart thermometer:

During the outbreak of a contagious disease, you start using a popular smart thermometer app. Apart from recording your temperature, the app allows you to input additional symptoms you might be experiencing. Based on your symptoms, you receive suggestions for actions you should take to protect yourself and others in the community from the contagious disease. The app uses a combination of Bluetooth and location data to measure exposure to the disease in communities. The app allows the data to be accessed by the authorities, doctors, and scientists so that it can be used to track the spread of the disease and enforce people’s compliance with containment measures, such as quarantines. The app continues to operate in the same manner even after the disease outbreak has dissipated.

We created the scenario in accordance with best practices for scenario-based research, such that it was open to interpretation, realistic, and did not elicit “right or wrong” responses [79]. The semantic content of the scenario was iteratively developed by both authors based on analyses of existing smart health devices (eg, thermometers, glucose monitors) that may be reasonably paired with contact tracing apps. We piloted the scenario with several individuals of diverse backgrounds, including different native languages, professions and education levels, and genders and ages. While changes based on the pilots were minimal, they helped us refine word choices and sentence structure for improved clarity and comprehension of the scenario.

The resulting scenario satisfies Meinert’s [79] best practices for scenario development by being realistic: Several smart thermometer apps are available to end users and are easily discoverable through any search engine. Each of these real-world, smart thermometers is paired with a smartphone-based app that stores and analyzes the data collected using the smart thermometer. We informed participants that the fictional smart thermometer app described in the scenario was “popular.” The provision of such information adheres to Meinert’s [79] best practices for scenario development relative to realism and openness to interpretation.

We added information on the app’s use of Bluetooth and location data to satisfy the openness to interpretation criterion of scenario development [79]. We further described data collected by the app as being available to a vague set of actors including “the authorities, doctors, and scientists.” The inclusion of such information additionally satisfies the realism requirement: It is common to describe pandemic tracking apps as using location data (eg, [80]) that, according to prior work on hyperbolic scaling [66] and digital resignation [28], can be expected to be accessed by unknown third parties. Empirical work has similarly shown that mHealth apps often do not present transparent or full accounts of third parties to whom data access may be granted [34].

Further, the scenario referenced the continued functionality of pandemic tracking apps after the pandemic has been controlled. The inclusion of this information is in line with current trends in mHealth research that examine the usefulness of pandemic tracking apps for postpandemic monitoring of health conditions (eg, [40]). We included the information in order to engage participants in a discussion of the long-term implications of digital contact tracing.

### Recruitment, Participants, and Interviews

We recruited participants through ads posted on Craigslist, Reddit, Facebook, and LinkedIn. Recruitment was entirely online due to restrictions on in-person research activities during the COVID-19 pandemic. The advertisements directed interested individuals to a screening questionnaire that asked for basic demographics (eg, age, gender, employment status, duration of residence in the United States). The complete screening questionnaire is available in Multimedia Appendix 1.

We used the responses to the screening questionnaire to compose a diverse sample. Given the exploratory nature of our study, we strove for a diverse pool of interviewees to explore as much of the terrain as possible. The sample diversity helped us collect rich data that include multiple perspectives [73].

We invited the selected individuals to participate in one-on-one interviews for which participants were compensated US $10. We specifically chose the one-on-one format to be sensitive to participant comfort given the likelihood that participants would discuss sensitive information (eg, personal experiences with COVID-19, privacy concerns) [81]. All interviews took place over Zoom and lasted between 45 minutes and 60 minutes. During the interviews, participants discussed several separate scenarios, including the one described in the earlier section. We ensured that participants understood the fictional nature of the scenarios. We included several scenarios to develop an understanding of privacy concerns across a wide range of apps. Participants were free to spend as long as they wanted to answer questions related to each of the scenarios. The portions of the interview guide pertaining to the smart thermometer scenario are available in Multimedia Appendix 2.

Table 1 presents an overview of participant demographics. Of the 19 participants, 9 (48%) identified as female, 8 (42%) as male, and 2 (11%) as nonbinary. The median age of the participants was 30 years. Of the 19 participants, 5 (26%) were university students, while the remaining 12 (74%) were professionals in fields varying from web design to package handling.

**Table 1:** Participant demographics.

Participant ID	Gender	Age (years)	Occupation
A	Female	25	Student (law)
B	Female	26	Web designer
C	Female	27	Student (human-computer interaction [HCI])
D	Nonbinary	21	Student (history)
E	Male	26	Writer
F	Male	29	Systems analyst
G	Female	23	Student (pharmacy)
H	Male	31	Student (informatics)
I	Female	26	Business analyst
J	Male	47	Call support
K	Female	25	Student (HCI)
L	Male	30	Software quality assurance
M	Nonbinary	30	Model
N	Male	36	Database admin
O	Female	42	Court clerk
P	Male	37	Sales executive
Q	Female	36	Homemaker
R	Male	36	Package handler
S	Female	46	Admin assistant

### Analysis

We analyzed the transcripts of the interviews using thematic analysis, adhering to the multistage process described by Braun and Clarke [75]. Initial engagement with the data took the form of repeated and collaborative readings of interview transcripts among 5 members of the research team. This phase culminated in open coding of the complete corpus of interview transcripts. The researchers then condensed the codes and identified emergent themes, arriving at a group of 5 initial themes. Upon further analysis, we reduced the initial 5 to the 3 themes we present in detail in the next section.

## Results

In this section, we present the results of the thematic analysis. We begin with findings surfaced through the consideration of pandemic tracking apps as part of a broader app-enabled health care system. We proceed to analyze the privacy concerns that emerge from the relationship between pandemic tracking apps and the broader ecology of smartphone apps to which they belong. We build upon this narrative by introducing and analyzing contradictory forms of the greater good that emerge from participant responses. Our findings highlight that perceptions of pandemic tracking apps indicate an expectation that such apps will form another brick in the wall of surveillance that people increasingly experience in their everyday lives.

### Public Health Versus Individual Privacy: The Perceived Trade- Off

Participants saw the use of pandemic tracking apps as a potentially effective way of interfacing with the health care system. For example, the fictional smart thermometer app described in the scenario was understood to serve a triage function that indicates when an individual should seek medical attention:

…it’s really important to resolve worries that people might have on a personal level, but then also if their fears are justified and they might have a disease or something like that. I think it’s also important for them to know that so that they can receive medical care or do what they need to do.

Participant D, nonbinary, 21 years old, student (history)

Participant D’s perception of the relationship between pandemic tracking apps and the broader health care system is generally positive. However, further analysis of this common sentiment expressed by several participants revealed a bigger picture. The entanglement of pandemic tracking app data with institutions, including but not limited to health care systems, implies the presence of unknown third parties who may gain access to user or patient data. Such privacy concerns are inherited from the broader app culture [66] and present users with difficult choices:

I’m feeling a bit conflicted. Part of me is like, “Okay, interesting.” Another part of me is like, “Oh, this is a little bit like Big Brother.”

Participant B, female, 26 years old, web designer

People routinely expect apps to collect data about them and to share those data with third parties [66] as part of surveillance capitalism [14]. Through Participant B’s invocation of “Big Brother,” it is apparent that pandemic tracking apps are not an exception to this expectation. Participants were concerned about who would have access to the data generated by pandemic tracking apps during, as well as after, the pandemic. They were similarly concerned about what those unknown third parties might do with the data:

I think just being aware of who’s using it, where is it being used, and how they’re using it is one thing. Like, what are their practices, who is using it, and what company is it being used for? Is it being used by just health workers? Is it being used to be able to track and sell information to further recommend products to us?

Participant K, female, 25 years old, student (HCI)

Indeed, supported by the implicit connectivity of pandemic tracking apps, each participant raised concerns about who would ultimately gain access to any data generated and collected by these apps. Participants raised similar concerns about the kinds of data that might be generated, collected, and stored. Such concerns ultimately frame the deployment of public health surveillance infrastructure built on the back of pandemic tracking apps as a “slippery slope”:

An acceptable purpose to me would be just to gather data on the disease and how it behaves in different communities and stuff. A malicious purpose would be them using this information to figure out where you live or more information about you, such as your age. Just in any way revealing your identity would be, to me, way more on the malicious side, although it’s not necessarily malicious outright. But to me, it’s a slippery slope.

Participant F, male, 29 years old, systems analyst

Participants further demonstrated the belief that data collected by contact tracing apps could be shared for profit with advertisers or as-yet-unknown third parties:

They could probably give it to advertisers or other people that I probably don’t want it to go into the hands of. ...I can’t think of a reason why it would be bad, but it just sounds bad, you know? It sounds sketchy. I don’t know what advertisers could use other than for advertising like vitamin C or other get-well things. But either way, it just seems bad.

Participant G, female, 23 years old, student (pharmacy)

In discussing concerns that public health apps would share user data with unknown third parties, participants referred to a category of unknown third parties that would gain access to data, but rarely identified specific actors who might belong to that category. Instead, participants expressed the belief that public health apps would work “too well,” thus facilitating the trafficking of data to unknown parties:

…it’s just knowing that the application is working and doing its thing, but potentially it’s working too well and that power getting into the hands of individuals that weren’t necessarily designed to have that information to begin with.

Participant G, female, 23 years old, student (pharmacy)

In considering the routinization of pandemic tracking apps as infrastructure for postpandemic public health surveillance, participants were faced with a difficult and contradictory set of possibilities. In particular, we observed a fundamental perceived trade-off between personal privacy and public health. This core trade-off appeared as an either/or condition: People can expect either personal privacy or better public health. Achieving both simultaneously did not seem realistic to participants. Moreover, participants seemed to place values in a hierarchy wherein public health was superior to personal privacy:

I value public health and public safety over personal privacy.

Participant A, female, 25 years old, student (law)

Participants routinely reported such higher consideration for public health over personal privacy even when expressing deep wariness regarding existing pandemic tracking infrastructure:

Google and Apple have added their tracing APIs to their operating system so that governments and health authorities could start pushing out these apps that do this tracing to track down where you’ve been and who you’ve been around to understand contagious disease, disease control, and all that stuff. I understand the benefit of it. But it’s highly intrusive, right? It’s tracking all your data, it’s tracking who you’re coming in contact with, it’s tracking your location. It’s kind of Big Brother-ish, in a way.

Participant G, female, 23 years old, student (pharmacy)

This quote from Participant G demonstrates that people are aware of pandemic tracking apps as they exist in the real world but generally hold an inaccurate understanding of their functionality. Participants expected pandemic tracking apps to trade identifiable data, even though the Apple/Google collaboration in the United States goes to great lengths to maintain user anonymity. Such inaccurate understanding likely arises from the pervasiveness of apps that use the sale of access to data as a primary economic motive [14,66]. However, that which is perceived to be real is real in its consequences. As such, when understanding people’s perceptions and expectations of routinized pandemic tracking apps, our focus is the way in which people believe such apps to work, regardless of whether the actual functionality matches these beliefs [82].

The perception of pandemic tracking apps as inherently privacy-intrusive constitutes a core component of the discourse of such apps. Indeed, being wary of the privacy implications of pandemic-tracking apps has real-world consequences:

You said that you saw someone voicing some concerns. What was that concern?

Interviewer

Oh, this is somebody that I personally know, and she kind of is an outlier in terms of her thinking. But she had something on there. She knew these apps, she had named some, and I did not. I have not heard of the specific names of the apps, but she had some of the apps listed, and she said that if you have any of these apps and you’re thinking about using them too, please delete me from your contacts, because I will not be traced and all this kind of stuff. It was kind of really out there.”

Participant O, female, 42 years old, court clerk

Despite general wariness and expectations that pandemic tracking apps will violate privacy by virtue of their connectivity with unknown third parties, participants routinely justified the necessity and use of pandemic tracking apps by referring to a nebulous concept — the greater good:

I’m fine with [using the app] for this particular reason, just because it’s for the greater good of society to know who’s sick and who could potentially be sick. I think that something that could help a lot of people and save lives kind of outweighs your right to privacy in some ways, so I don’t have a problem.

Participant O, female, 42 years old, court clerk

As we describe in the next subsection, participants routinely employed “the greater good” as a means of justifying or overlooking the perceived long-term privacy concerns raised by pandemic tracking apps. Yet, we found that this greater good is multifaceted, and the relationship between the different facets of the greater good is contradictory, highlighting pandemic tracking apps as a potential site for surveillance creep: the colonization of health by the economics of surveillance capitalism [14].

### Rationalizing the Health/Privacy Trade-Off With the Greater Good

Pandemic tracking apps exist within a wider ecology of apps. Therefore, privacy concerns that emerge from user interactions with this wider ecology color perceptions and expectations of pandemic tracking apps [66]. When pandemic tracking apps are expected to align with the economic practices of surveillance capitalism (ie, monetizing user data by selling it to third parties), privacy concerns about pandemic tracking apps take center stage. Such concerns include wariness regarding unknown third parties who may gain access to the data generated, collected, and stored by pandemic tracking apps and inaccurate, but nonetheless meaningful and deep-seated, perceptions of how such apps function. The greater good is the primary means by which people rationalize and justify engaging in what they perceive as the inherently privacy-affecting decision to use pandemic tracking apps. The use of the greater good to rationalize the adoption or routinization of pandemic tracking apps can be rather sharp, demonstrating frustration with the complexity of issues raised by such apps:

But at the same time, yeah, actually no ... I think it’s fine because it just seems to take small vitals like temperature, and if they’re probably sick, it might be fine. We’ll say it’s fine because it’s for the greater good.

Participant G, female, 23 years old, student (pharmacy)

This excerpt depicts a trade-off in motion. Participant G quickly reverted to the greater good as a means of rationalization. She vacillated between further expressing her concerns about the fictional smart thermometer app — “but at the same time, yea, actually no” — and finding an easier conversational way out of such expression. Participant G’s use of the phrase, “We’ll say it’s fine,” indicates a clear tension: the implicit recognition of privacy-related problems and the desire to achieve the greater good despite them.

The scenario suggested that pandemic tracking apps may be used as a foundation for postpandemic public health monitoring. Participants generally reacted negatively to this possibility:

It would be most useful for you to share everything or as much as you can during a pandemic, but outside of a pandemic, it starts to be kind of a gray area.

Participant F, male, 29 years old, systems analyst

Invocation of the greater good is not sufficient to rationalize the long-term implications of pandemic tracking apps should their use be routinized as a means of health surveillance in general. The greater good that pandemic tracking apps serve appears to have a shelf life:

So, during the outbreak of a contagious disease, I would think it’s worth it to give up privacy and data and information. If it’s an app that will really help and literally save lives, I’d be happy to do that. But I don’t like the last part that it continues to operate in this way after the outbreak has dissipated.

Participant A, female, 25 years old, student (law)

The greater good appears to refer specifically to the mitigation and control of the pandemic, but does not necessarily extend to the routinization of pandemic tracking apps as a form of postpandemic public health surveillance:

Obviously when there’s an outbreak of a contagious disease, everyone’s focus is really just on that. Now, of course, you never know that 100%. Other people can still utilize that data for malicious reasons or their own reasons. But you would think that there’s something bad going on there. Everyone’s trying to help as much as possible. You’re okay with it. You’re okay with exposing yourself in this way. It’s just the app being able to be used in the same manner after disease outbreak has dissipated … that part is more uncomfortable.

Participant L, male, 30 years old, software quality assurance

It is not, however, Participant L’s expected or experienced discomfort that takes the center stage. Rather, it is what such discomfort implies. While our scenario intentionally primed participants to consider postpandemic routinization of pandemic tracking apps, the phrasing was ambiguous as to postpandemic functionality of such apps. We allowed participants to extrapolate from their contemporary perceptions and expectations of pandemic tracking apps in order to understand what routinization of such apps might mean.

When read in terms of people’s expectation that data collected by pandemic tracking apps will be shared with third parties for financial gain, Participant L’s discomfort signals the expectation that the apps will contribute to a broader culture of surveillance. Yet, participants generally accepted the likelihood of such apps being routinized for public health surveillance after the pandemic, with phrases such as, “We’ll get to that when we get to that.”

It’s just the app being able to be used in the same manner after disease outbreak has dissipated…that part is more uncomfortable because it’s unnecessary information being exposed when it doesn’t really need to be unless the app can sort of justify it, right? If it’s justified and valid, let’s say let’s see. Doctors and scientists and authorities. Who are you? Well, I don’t know who the authorities are, but I guess it is the police. I don’t know. I guess we’ll get to that when we get to that.

Participant L, male, 30 years old, software quality assurance

Taken together, participant discussion of the greater good and their willingness to forego privacy concerns for that greater good can be troubling. Even though it is defined only implicitly, the greater good becomes a floating signifier used to justify and rationalize the perceived privacy risks of contact-tracing applications:

The app continues to operate in the same manner even after the disease outbreak has disappeared? Doing something for the greater good always gets my vote. So, exposing my information like this, if it’s toward a greater good like this, I will tell you, again, like I sort of mentioned before, I know what’s happening.

Participant L, male, 30 years old, software quality assurance

People understand and even expect privacy breaches as a result of app-based contact tracing. Further, they see the possibility of “Big Brother” levels of surveillance as real. The invocation of “Big Brother” refers to the expectation that contact tracing apps will result in heightened surveillance. That is, people perceive that the routinization of pandemic tracking apps for public health surveillance will lead to oversurveillance by a heterogeneous set of actors (eg, technology corporations, government bodies, health care organizations, insurance providers, third-party advertisers) variously responsible for app maintenance, data analysis, public health regulation, and health care delivery. This amounts to a future that is not accurately characterized by widespread positive affective experience despite that future’s roots in the greater good:

What do you imagine the enforcement with compliance could mean?

Interviewer

Nothing that I want to imagine in real life.

Participant M, nonbinary, 30 years old, model

Participant M implies that public health surveillance achieved through the routinization of pandemic tracking apps would lead to outcomes too negative to even imagine. Such expectations directly call into question the short- and long-term forms of the greater good to which adoption of pandemic tracking apps contributes. On one hand, the adoption of pandemic tracking apps can contribute to the greater good of public health during a pandemic. On the other hand, the long-term adoption of such apps as infrastructure for public health surveillance contributes to widespread concerns of mass surveillance and privacy violations that cannot reasonably be described as a greater good. In fact, the privacy ramifications could arguably erode the greater good.

## Discussion

Two facets of our findings are particularly worthy of discussion. First, we describe the benefits and necessity of taking a long-term, sociotechnical approach to understanding the potential routinization of mobile apps as part of the public health infrastructure. Second, we describe how user privacy concerns constitute a productive means for identifying long-term implications of an emergent class apps used for public health surveillance.

### People Expect Privacy-Related Infrastructural Problems in Public Health Surveillance

Direct consideration of people’s privacy concerns about pandemic tracking apps indicates their wariness about the routinization of such apps as infrastructure for postpandemic public health surveillance. Although users are willing to justify the use of apps that raise privacy concerns with the concept of the greater good, such justification has a limited shelf life. By stating expectations that privacy concerns related to pandemic tracking apps extend to mHealth apps in general, participants highlighted the need to decouple or disentangle mHealth infrastructure from the privacy-invasive practices of surveillance capitalism.

People are wary of pandemic tracking apps because they might “work too well” (Participant G) and therefore demonstrate hyperfunctionality as identified by Seberger and Bowker [71]: conditions wherein infrastructures function within the boundaries of their designed functionality but do so in a way that gives rise to unexpected outcomes. In other words, if pandemic tracking apps achieve the form of control over the spread of the pandemic that they are designed to achieve, such success might spill over to other uses. In our scenario, we pointed out the potential for such other uses in the form of postpandemic public health surveillance that uses such apps as its infrastructure.

Participants routinely assumed that data collected via pandemic tracking apps would be shared with an identified-but-unknown category of third parties. We interpret participant concerns over third-party access to data collected by pandemic tracking apps as a form of hyperbolic scaling [66], where concerns regarding privacy violations transfer from known apps to new apps. In this logic, if App A (a known app) demonstrates problematic functions relative to end-user privacy, then *all* apps are assumed to demonstrate the same characteristics.

### Stakeholders Should Take a Long-Term View of Public Health Surveillance

A pandemic often affects people’s daily lives and routines in profound ways. Because the conditions of a pandemic can be all-encompassing, it can be difficult to take a long-term perspective. Yet infrastructure, like surveillance, creeps. It is learned and normalized through use [37]. The misalignment between short- and long-term concerns is compounded by the immediacy of the language used to communicate and frame the threats of the pandemic [83]. With visions of large-scale pandemic tracking already being considered (eg, [6,40-42]), it is poised to become infrastructure for public health surveillance. We suggest that it is necessary to understand the impact of public health surveillance at a temporal scale aligned more closely with the long-term one of infrastructure [84]. People’s judgments related to trade-offs that unfold at the scale of the short- and long-term are notoriously problematic [85]. The severity of the pandemic and its social effects emphasize the urgency of triage to fix the most immediate problems first by focusing on the greater good of public health. Yet, given the combined power of medicine and technology to achieve social change, it is not entirely appropriate to focus solely on the immediate threats posed by the pandemic. The immediate actions that governments, corporations, and institutions take in service of pandemic mitigation will reverberate in the sociotechnical structures of the postpandemic world [86].

People’s perceptions of a core trade-off between individual privacy and public health highlight a need for long-term thinking about public health surveillance. People are willing to forego privacy concerns for the greater good even though they are uncomfortable with surveillance by the technologies used to achieve that greater good. Our study therefore supports the findings of prior work on the mitigating effects of health concerns on people’s privacy concerns [87]. We provide further support for findings derived from a study with UK participants who saw the greater good as a motivating factor for app use [7]. We extend the work of Williams et al [7] by adding practical nuance to the role that the greater good plays in pandemic mitigation and surfacing the potentially slippery slope toward increased and routinized public health surveillance.

The likely impacts of app-based public health surveillance extend beyond public health itself. This extension includes potential impact on people's expectations of privacy as well as colonization of mHealth by the economics of surveillance capitalism. Such colonization may lead to diminished trust between patients and health care providers. Given people’s deep-seated privacy concerns, app creators, policy makers, and health institutions should engage in detailed and transparent public relations work to communicate the ways in which user privacy is protected. Such communications should describe exactly which third parties, if any, will be given access to data collected through apps used for public health surveillance and under what circumstances. While privacy concerns raised by pandemic tracking apps are likely as widespread as the pandemic itself, the steps described should be taken within existing local legal frameworks (eg, Health Insurance Portability and Accountability Act [HIPAA], General Data Protection Regulation [GDPR]). Overall, our insight highlights the need for an interdisciplinary approach to the development and deployment of app-based public health surveillance.

### It is Necessary to Disentangle Public Health Surveillance From Surveillance Capitalism

People’s perceptions of the alignment between public health apps and the economics of surveillance capitalism may have a detrimental effect on the relationship between patients and health care systems. Trust between patients and health care providers is foundational to effective medical care [88]. As such, the enrollment of patients as users in a sociotechnical system contextualized by potential mistrust serves neither the patient nor health care professionals. In a way, it breaches the core medical credo of “first do no harm.”

Users are inherently vulnerable to the power structures instantiated by the apps that they use [66]. But, when users are also patients, they are doubly vulnerable: vulnerable to the conditional empowerment implicit in app use [66] and vulnerable to the loss of control that comes with the status of being a patient [89] (perhaps they are also vulnerable to the dehumanization of data-driven representation [27]). If pandemic tracking apps achieve the status of infrastructure — invisibility through successful deployment and adoption [37,38] — they may do so at the risk of solidifying the vulnerability-inducing conditions of surveillance capitalism within a medical context [14]. Such solidification would directly contrast with the notion of care central to medical practice. In this light, a broader, socially oriented approach to the medical credo, “first do no harm,” would benefit end users of pandemic tracking apps.

We contend that people’s expectation of, and resignation to, being used may foreshadow the colonization of public health surveillance by the data-hungry and profit-driven mechanics of surveillance capitalism. We further contend that this is as much a public health issue as the maintenance of more narrowly defined bodily health. We therefore suggest the development of public health policies that account for the long-term sociotechnical effects of public health surveillance. Such policies, developed within the legal frameworks of specific regions, would necessarily view patients not solely with the medical gaze, but through a more sensitive lens that considers end-user empowerment, trust in digital systems, and the emerging broader relationship between health care and surveillance capitalism.

We further suggest that the activation of infrastructure for public health surveillance be legislatively limited to times of scientifically defined public health crises. It would be unwise to discard infrastructure that’s already been built. The Google and Apple collaboration in the United States, for example, likely constitutes a useful resource for pandemic preparedness. However, such infrastructure does not necessarily present an ethical or beneficial means of general public health surveillance, given the profound privacy concerns about pandemic tracking apps. The routinization of pandemic tracking apps as infrastructure for public health surveillance has the potential for profound negative impact on the trust that should characterize the relationship between patients and health care providers. We found that people believe that health surveillance apps will operate according to the economics of surveillance capitalism, wherein access to data is sold for profit, typically without much regard for user privacy concerns. When people believe that health surveillance apps align with surveillance capitalism, it is unreasonable to expect them to trust the system that profits from the sale of their data. Such a dynamic sets up the potential for such apps to diminish patient trust in the health care systems that ostensibly care for them.

Given the dangers present in the perceived alignment between app-based public health surveillance and surveillance capitalism, we suggest that the activation of public health surveillance be limited to populations that meet specific criteria. Such criteria might include population density, smartphone saturation, and rate of disease transmission. In this way, an app for public health surveillance might be utilized to the greatest effect, while minimizing the potential for surveillance creep. Beyond meeting well-defined medical standards, such population-specific deployment should be overseen by multidisciplinary teams capable of assessing the sociotechnical and surveillance-related impact on populations who are already, by virtue of an infectious agent, fundamentally at risk.

### Limitations and Future Work

We collected data during a specific period from a relatively small group of participants. Since the COVID-19 pandemic is still ongoing, additional studies are needed to investigate how the trajectory of the pandemic might influence these matters. Public health and privacy concerns are inherently cultural. Further studies involving participants from other populations would add greater context to our findings. In particular, it is possible that our findings may be specific only to the United States. Many social norms and expectations in the United States differ from those in other countries. In the United States, perceived economic relationships between government agencies, health care providers, and for-profit corporations (eg, app makers, health insurance companies) create a structure in which people may expect their privacy to be breached. Such relationships qualify people’s trust (or lack thereof) in government that may be different than that in other regions across the world. Nonetheless, our findings highlight the need for further work regarding the role of trust in institutions and the possible success of app-based public health surveillance after the pandemic has ended. Further work is required to understand perceptions and expectations of public health apps in other cultural contexts.

Given the exploratory nature of this work, we did not consider ethnicity as a factor in our sample construction. However, when choosing interviewees from a pool of eligible participants, we did strive for diversity in ages, genders, professions, and educational backgrounds. Prior work [7] has not analyzed the effect of ethnicity either nor can a qualitative study of limited sample size such as ours yield robust findings relative to such a sensitive topic. That said, we recognize the profound importance of ethnicity in matters pertaining to the greater good and identify this as an important area for further research.

### Conclusion

Given that pandemic tracking apps have been promoted as a useful tool for pandemic mitigation, it is likely that such apps will form the backbone of postpandemic infrastructure for public health surveillance. While it is clearly important to understand the short-term, pandemic-specific privacy concerns that arise from such apps, we contend that it is equally important to understand their long-term sociotechnical implications. People frame the use of pandemic tracking apps in terms of achieving a greater good: acceptable levels of public health. However, such framing is contextualized by a core trade-off with individual privacy. The trade-off reveals a deep-seated tension in the use of the greater good rationalization: the perception that pandemic tracking apps implicitly align with the data-hungry economics of surveillance capitalism. To make matters worse, people are resigned to viewing such alignment as inevitable, which may harm their trust in the institutions that provide health care. We highlight the relevance of analyzing privacy concerns when considering the potential routinization of apps for public health surveillance and call for the multidisciplinary application of medically influenced ethics in the design, development, deployment, and data use of mHealth apps designated for everyday use.

## Appendix

Multimedia Appendix 1 Screening questionnaire.

Multimedia Appendix 2 Scenario.

## Abbreviations

GDPR: General Data Protection Regulation
HCI: human-computer interaction
HIPAA: Health Insurance Portability and Accountability Act
mHealth: mobile health
