# Supplementary material for: Post-COVID Public Health Surveillance and Privacy Expectations in the United States: Scenario-Based Interview Study
Source: JMIR Mhealth Uhealth. 2021 Oct 5;9(10):e30871. doi: 10.2196/30871 (PMC8494069; doi:10.2196/30871)
Supplement: Multimedia Appendix 1 [file mhealth_v9i10e30871_app1.docx]

### Screening Questionnaire

Study Information & Demographic Questions

Thank you for your interest in participating in our study on People’s Perceptions of Technology-Related Scenarios.

Please fill out this brief one-minute questionnaire about yourself. We will use your answers to determine if you are eligible to participate in the study.

If you qualify, we will contact you via email for a 45- to 60-minute interview.

As a token of our appreciation for your participation in the interview, you will receive $10 cash or cash equivalent, such as an Amazon gift certificate. If you do not qualify for participation, your responses will be safely discarded.

- What is your year of birth?

[dropdown menu of years from 1900-2020]

- What is your gender?

- Male

- Female

- Non-binary

- Prefer to self-describe: [text box]

- Prefer not to say

- How long have you lived in the United States?

- All my life

- Less than 1 year

- 1 year

- 2 years

- 3 years

- 4 years

- 5 years

- 6 years

- 7 years

- 8 years

- 9 years

- 10 years

- More than 10 years

- What is the highest level of education you have completed? (If currently enrolled, choose the highest degree received.)

- Less than high school

- High school

- Vocational training

- Some college

- College graduate (B.S., B.A., or other Bachelor’s degree)

- Master’s degree

- Doctoral degree

- Professional degree after college (e.g., law or medical school)

- Something else. Please specify: [text box]

- Are you a resident of Bloomington, Indiana?

-Yes

-No

- Are you affiliated with Indiana University?

-Yes

-No

- (If affiliated with Indiana University) What is your affiliation with Indiana University? (*Check all that apply.*)

- Undergraduate student

- Graduate student

- Faculty

- Staff

- Retired

- Something else. Please specify: [text box]

- (If undergraduate or graduate student) What is your major/field of study?

[text box]

- (If faculty or staff or retired) Which department or school are you affiliated with?

[text box]

- If you qualify for the study, which email address should we use to contact you for scheduling an interview?

[text box]
