# Supplementary material for: Post-COVID Public Health Surveillance and Privacy Expectations in the United States: Scenario-Based Interview Study
Source: JMIR Mhealth Uhealth. 2021 Oct 5;9(10):e30871. doi: 10.2196/30871 (PMC8494069; doi:10.2196/30871)
Supplement: Multimedia Appendix 2 [file mhealth_v9i10e30871_app2.docx]

### Scenario

“During the outbreak of a contagious disease, you start using a popular smart thermometer app. Apart from recording your temperature, the app allows you to input additional symptoms you might be experiencing. Based on your symptoms, you receive suggestions for actions you should take to protect yourself and others in the community from the contagious disease. The app uses a combination of Bluetooth and location data to measure exposure to the disease in communities. The app allows the data to be accessed by the authorities, doctors, and scientists so that it can be used to track the spread of the disease and enforce people’s compliance with containment measures, such as quarantines. The app continues to operate in the same manner even after the disease outbreak has dissipated.”

### Semi-Structured Interview Guide

- What do you think about this scenario?
- Do you use any apps connected to smart devices, such as a smart thermometer, a smart speaker, a fitness tracker, etc.? Why or why not?
- How do you feel about providing your symptoms to the app described in the scenario?
- How do you feel about receiving personalized health guidance based on your symptoms?
- How do you feel about the use of your data for tracking the health of your community?
- How do you feel about the app data being accessed by the authorities, doctors, and the government?
- What would make you more or less likely to favor such data access?
- For what purposes, if any, should the authorities, doctors, and government use the data?
- How do you feel about the enforcement of quarantines based on the data collected by the app described in the scenario?
- If the participant expresses negative (or positive) opinions of ads or advertising, then ask why.
